# Crag Martin neontology complements taphonomy at the Gorham's Cave Complex

**DOI:** 10.1038/s41598-021-95974-9

**Published:** 2021-08-19

**Authors:** Keith Bensusan, Tyson Lee Holmes, Charles Perez, Geraldine Finlayson, Stewart Finlayson, Rhian Guillem, Clive Finlayson

**Affiliations:** 1 Institute of Life and Earth Sciences, University of Gibraltar, Botanic Gardens Campus, PO Box 843, Gibraltar, GX11 1AA Gibraltar; 2 Gibraltar Ornithological & Natural History Society, Jews’ Gate Field Centre, Gibraltar, GX11 1AA Gibraltar; 3 Institute of Life and Earth Sciences, University of Gibraltar, Gibraltar National Museum Campus, 18-20 Bomb House Lane, Gibraltar, GX11 1AA Gibraltar; 4 Department of Life Sciences, Liverpool John Moores University, Liverpool, UK; 5 Department of Anthropology, University of Toronto Scarborough Campus, Toronto, Canada

**Keywords:** Behavioural ecology, Behavioural ecology

## Abstract

Species present in the fossil record may continue to exist at an archaeological site, allowing study that fine-tunes our picture of the ecological past. A large wintering population of Eurasian Crag Martins *Ptyonoprogne rupestris* (ECM) roosts at the ‘Gorham’s Cave Complex’ UNESCO World Heritage site in Gibraltar, which is best known for its occupation by Neanderthals at times when ECMs were also present. Its complex geomorphology allows the study of use of different micro-sites (caves) within the roost. We used mark-recapture to test whether birds showed fidelity to micro-sites for roosting, and for differences in condition of birds across micro-sites. ECM showed very high fidelity towards micro-sites, within and between years, with > 90% chance of recapture at caves where they were first caught. Condition of birds differed between micro-sites, suggesting differences in roost quality between caves; birds were more likely to be recaptured at the micro-site where birds were in best condition, indicating higher survivorship. Our results demonstrate extremely fine-scale fidelity at the largest roosting site documented for ECM globally. Implications for conservation are discussed. The study provides current knowledge of a bird that has been using these caves since the Pleistocene and more generally on these caves as refuges.

## Introduction

An advantage of archaeological sites is that they offer opportunities to reconstruct past ecological communities^1^. Though used less often, the study of current processes at such sites can provide information on the ecology of species present in archaeological sites^2^, enriching our understanding beyond the information provided by taphonomy. Here we argue that the neontological perspective to ecology is a separate tool to the traditional palaeoecological approach, and is a powerful weapon in support of taphonomic analyses and interpretations. We use the Eurasian Crag Martin *Ptyonoprogne rupestris* (ECM) at the Gorham’s Cave Complex, UNESCO World Heritage Site^3^, as a case study to illustrate the potential of the method.

ECM are distributed from Morocco and Portugal in the west to the Pacific coast of China in the east^4^. Although Asian populations are migratory, those around the Mediterranean Basin—which includes a large proportion of the European range—are largely sedentary, with birds from further north (such as the Alps and southern Carpathians) moving mainly to the Mediterranean region^4^ and Iberian birds from the peninsular interior moving south and towards the coasts^5^. Thus, the species is the only aerial insectivore for which much of the European Population winters around the Mediterranean^6^. It nests solitarily or in small colonies^7^ but during the winter it can form large roosts^4^, which presumably increases the likelihood of discovery of ECM fossil material.

Gibraltar’s fossil avifauna is well studied (e.g.^8–10^). The ECM has had a relationship with the archaeological site known today as the Gorham’s Cave Complex in Gibraltar for tens of millennia, at least since the Late Pleistocene^11^, suggesting particularly stable and optimal conditions for this species despite severe climatic fluctuations. The species is currently present at the site during the autumn and winter^12,13^, providing opportunities for research on the existing population that could shed light on past events and aid in the conservation of potentially vulnerable sites.

The winter is a potentially limiting period for the survival of populations of migratory birds wintering in temperate zones^14–16^ and many aspects of their ecology may impact on survivorship. Roost site selection can be expected to be crucial and fidelity to winter roost sites has been reported for a range of bird species^17–20^. However, our knowledge of winter roost behaviour in migratory birds needs to improve (e.g., hirundines^21^), particularly as some winter roosts are known to hold a large proportion of a species or population (e.g.^22^) and are therefore exceptionally vulnerable to contingent events.

Here we report, for the first time, detailed evidence that birds are not only faithful to winter roosts (both within- and between-years) but that they are also faithful to particular locations (micro-sites) within these roosts. The Gorham’s Cave Complex is estimated to harbour ~ 1–2% of the European population of ECM (more below). Our results are important to our understanding of the ecology of a species present at the site for millenia, as well as the conservation needs of communally roosting migratory bird species, which have typically received much less attention at their wintering quarters (e.g.^21^). They open an avenue for future research and also illustrate the value of interdisciplinary research. Viewed from the millennial perspective, ECM conservation at the Gorham’s Cave Complex is not just about the protection of a living species but is also about the conservation of ecological and eco-cultural processes that are very much a part of our universal patrimony.

## Results

**Figure 1:**
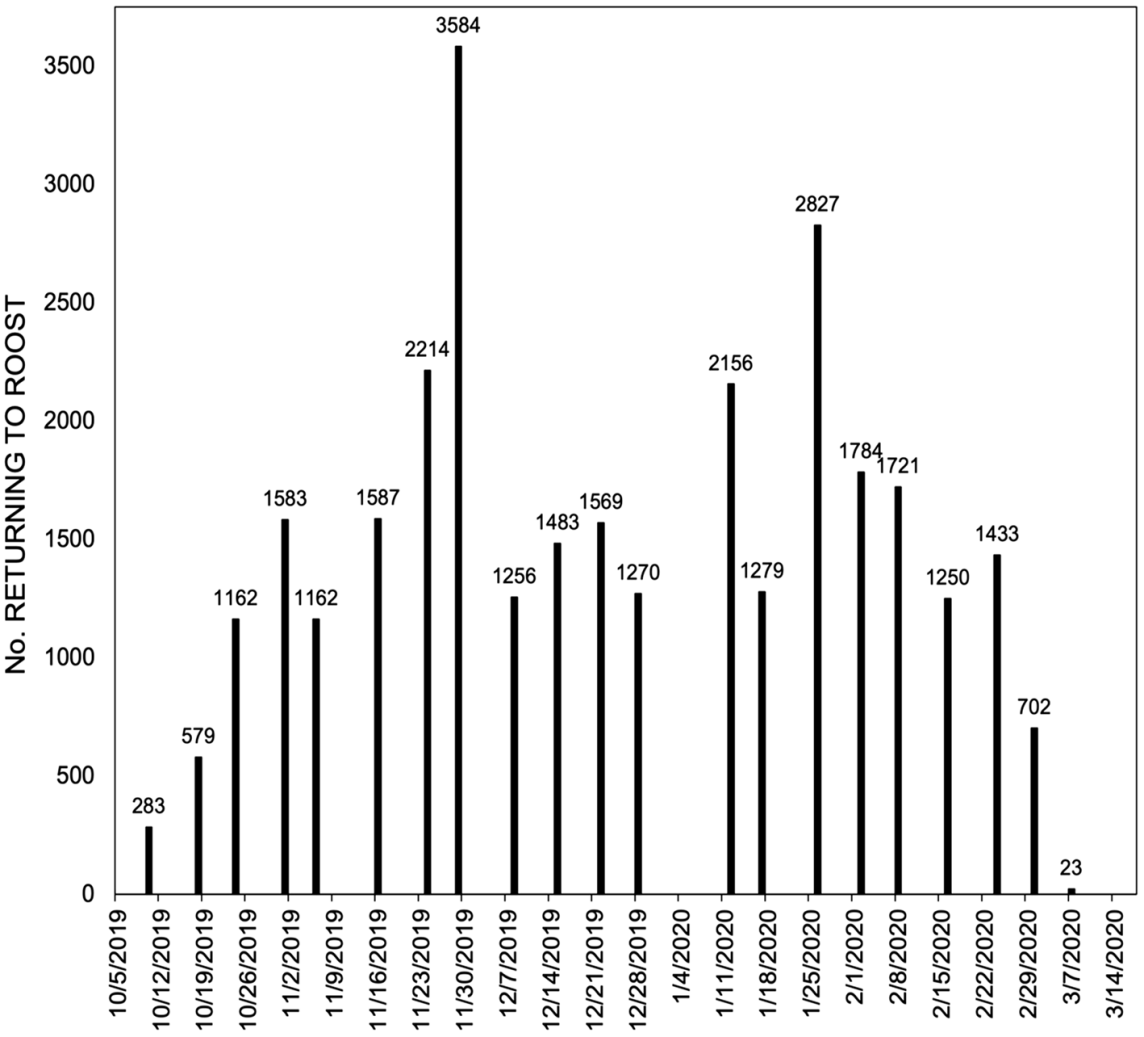
Weekly counts of Eurasian Crag Martins returning to roost at the Gorham’s Cave Complex, Gibraltar.

Weekly counts of ECM returning to their roosting site (see description of The Site below) during evenings showed considerable fluctuation between weeks (Fig. 1). ECM were recorded at the wintering site for approximately 154 days, within the period 10th October, 2019–12th March, 2020. Weekly counts fluctuated between 1162 and 3584 (mean = 1767, 155 SE) during the period that birds were assumed to be settled in the area, after migrants had moved on, between 1 November and 1 March. Furthermore, counts during the 2020-2021 season have exceeded these figures, with an exceptional 12,000 birds recorded in a single count. Mark-release-recapture data estimated that the number of birds using each of the three caves as winter roosts was 1285 in Vanguard Cave (95% CI 832, 2104), 998 in Gorham’s Cave (95% CI 817, 1281) and 677 in Cave F (95% CI 482, 984), for a total 2960 birds across the three caves (95% CI 2131, 4368). These results are therefore consistent with count data during the study period.

**Figure 2:**
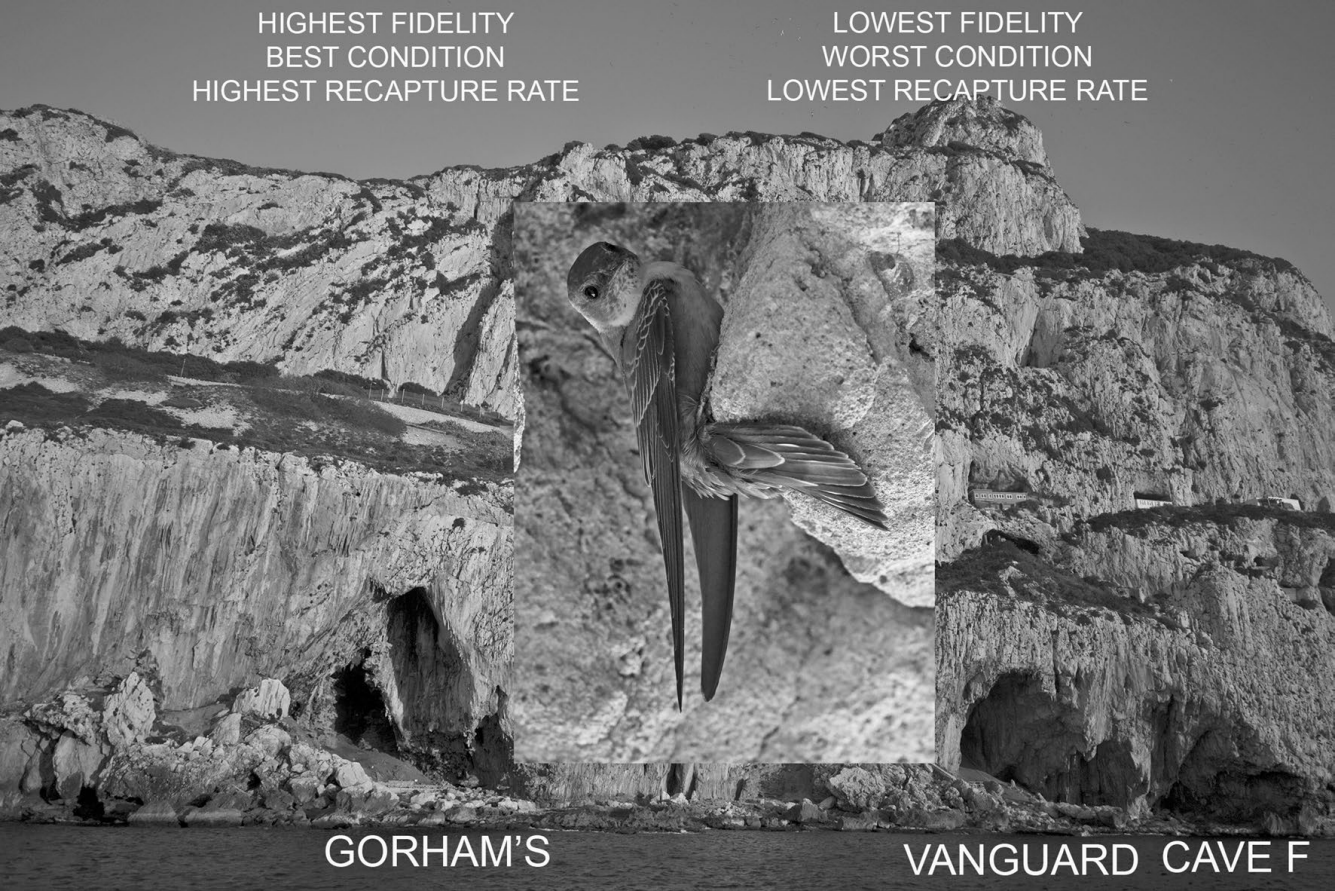
Degree of fidelity, condition and recapture rate of Eurasian Crag Martins across the Gorham’s Cave Complex, Gibraltar.

A high level of fidelity existed between the caves during the 2019–2020 wintering period (binomial test: *Z* = 8.16, *P* < 0.00000001), with the great majority of recaptures occurring at the cave where the birds were originally ringed (92 of 101 birds recaptured: a 91.1% chance of recapture at the cave where each bird was ringed). Furthermore, 37 birds were recaptured that had been ringed during separate seasons (10 Gorham’s Cave, 13 Vanguard Cave, 14 Cave F) and all of these were captured at the caves where they had originally been ringed (100% rate of recapture at the cave where each was ringed; binomial test: *Z* = 5.92, *P* < 0.00000001). Micro-site fidelity therefore held constant between years. There was also a significant difference in the strength of fidelity between caves within the roost during the 2019-2020 wintering period (Fisher’s exact: *P* 0.02), with birds initially ringed at Gorham’s Cave being captured less frequently in other caves than vice-versa (Table 1; 1.89% birds from Gorham’s Cave, 15.38% Cave F, 18.18% Vanguard Cave; Fig. 2). Therefore, micro-site fidelity was high across caves but highest at Gorham’s Cave.

**Table 1:** Biometric, recapture and age data (where ageing of individual birds was possible) for ECM at the three study caves. Wing length and weight are given 1 ± SE.

	Gorham's cave	Cave F	Vanguard cave
**Biometrics**			
Wing length (mm)	129.65 ± 0.15	129.83 ± 0.21	129.79 ± 0.20
Weight (g)	24.97 ± 0.10	24.33 ± 0.14	23.79 ± 0.14
**Recaptures**			
Recaptured same cave	52	22	18
Recaptured different cave	1	4	4
Recaptured once	34	17	18
Recaptured more than once	18	5	0
**Age**			
Adult	77 (35.3%)	16 (18.6%)	20 (22.0%)
Juvenile	141 (64.7%)	70 (81.4%)	71 (78.0%)

**Figure 3:**
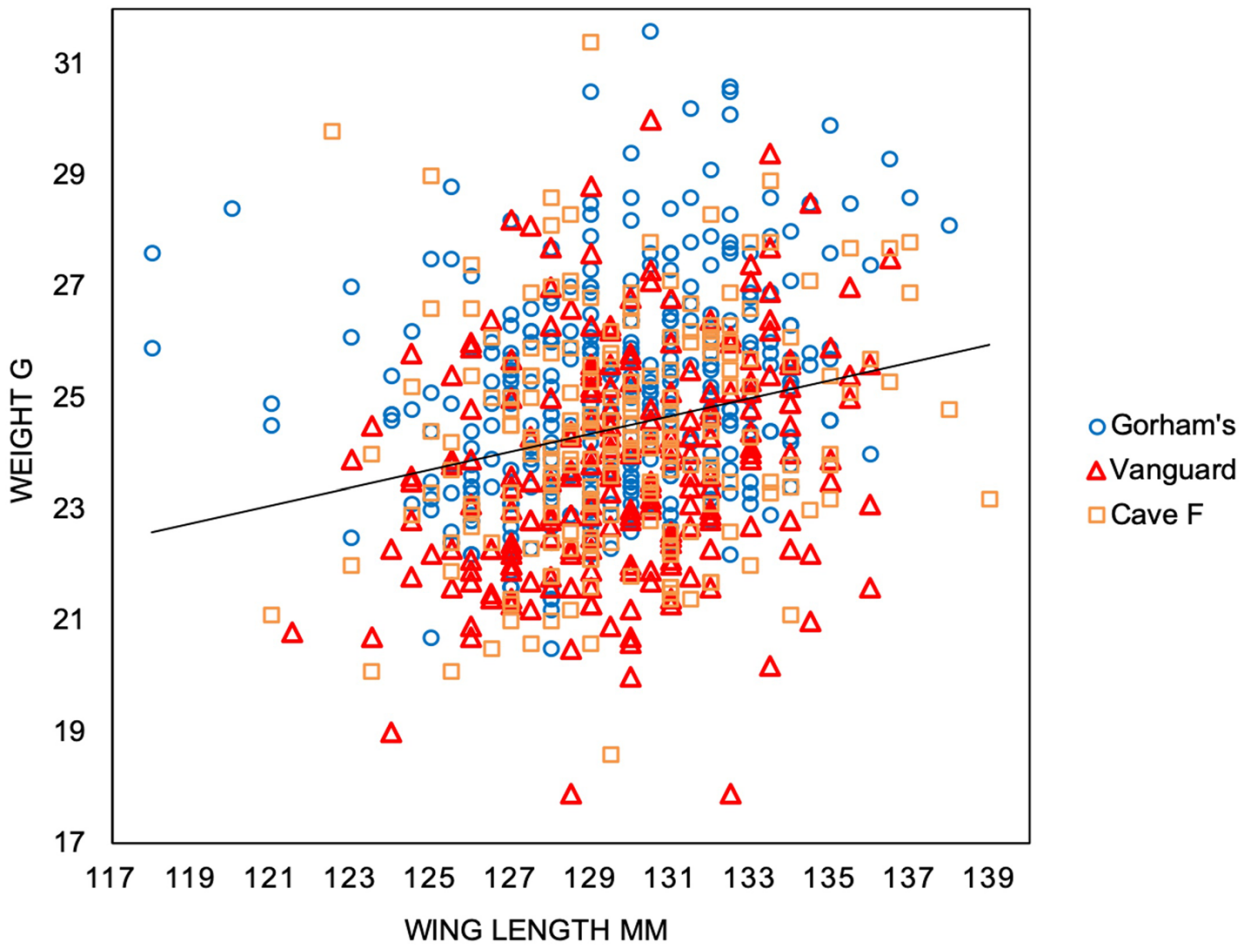
Relationship between weight (g) and wing length (mm) among Eurasian Crag Martins at the three caves used as winter roosting sites (*n* = 796, *R*^2^ = 0.05).

We found a positive relationship between weight and wing length in roosting ECM (linear regression: *y* = 3.72 + 0.16*x*; *F*_1,794_ = 45.40, *R*^2^ = 0.05, *P* < 0.00000001; Fig. 3). When residuals were grouped by cave, we found a significant difference between mean residuals generated for each micro-site, showing that birds were, on average, in best condition at Gorham’s Cave and worst at Vanguard Cave (one-way ANOVA: *F*_2,793_ = 28.16, *P* < 0.00000001; Table 1 for mean wing length and mean weight across caves; Fig. 2). Differences in weight and wing length have been demonstrated previously between adult and juvenile ECM in Gibraltar^13^. A significantly greater proportion of adults roosted at Gorham’s Cave than Vanguard Cave or Cave F (Pearson *x*^2^_2_ = 10.98, *P* 0.004; Table 1). These differences strongly suggest a difference in quality of roosting sites.

Daily recapture rate at all caves was related to number of days from the first day of trapping at each cave (linear regression: *y* = 0.015 + 0.003*x*; *F*_1,30_ = 18.71, *R*^2^ = 0.38, *P* < 0.0002). There was a significant difference between mean residuals generated for each cave, showing that recapture rates were highest at Gorham’s Cave, then Cave F, and lowest at Vanguard Cave (one-way ANOVA: *F*_2,29_ = 5.10, *P* 0.01; Fig. 2). The rate of recapture was therefore highest at the cave where the birds were in best condition and lowest at the cave where the birds were in worst condition. This indicates that survival among ECM was highest at Gorham’s Cave and lowest at Vanguard Cave, since the probability of recapture should be highest among birds that survive the entire winter. Furthermore, the likelihood of recapture more than once was greater at Gorham’s Cave than at the other two caves (Pearson *x*^2^_2_ = 8.62, *P* 0.01; Table 1; 34.62% birds from Gorham’s Cave, 22.73% Cave F, 0% Vanguard Cave). These tests together suggest a difference in winter survivorship between birds roosting at the three caves, with survivorship highest at Gorham’s and lowest at Vanguard.

## Discussion

Our results demonstrate that the Gorham’s Cave Complex remains of high value to a species that has been present at the site since the Pleistocene. Our results also show that the large numbers at the site, providing a source of skeletal material to the deposits even today, occur in the period October–March. The current paper provides, for the first time, fine-grained data enabling a better understanding of the ecology of the site and this particular species. Micro-site differences in species behaviour, between the different caves at the site today, reveal a complexity hitherto unappreciated from fossil evidence alone. Our results also offer the possibility to serve as a model for a new approach to the examination of other extant species with a similar chronological background, when these can be shown to have a sustained relationship with a particular site.

ECM showed an unexpectedly high level of fine-scale fidelity to their roosting micro-sites at the three caves today. The scale of fidelity observed is remarkable because the micro-sites lie at between 50 and 150 m from each other at their center points, within the same site and equally accessible to all birds in terms of distance involved when returning to roost. Data from birds recaptured in the three different sections of the roost (micro-sites) that had been ringed there during previous seasons strongly suggest that, not only do ECM return to roost in Gibraltar between years as had already been reported^13^, but the fine-scale fidelity to individual micro-sites is maintained to a large degree between years. This is the first time that such fine-scale fidelity to winter roosts has been recorded in a communally roosting passerine.

The condition of birds differed between the micro-sites, with birds in best condition at Gorham’s Cave and worst at Vanguard Cave. This suggests a difference in the quality of each cave as a refuge, which could be the driver of micro-site fidelity. Benefits that are thought to underlie communal roosting in birds include a reduction in thermoregulatory demands, a decrease in predation risk, and an increase in foraging efficiency^23,24^. The latter is an unlikely factor for differences observed among ECM in Gibraltar because they roost at a single site, departing for their foraging grounds and arriving to roost together. Peregrine Falcons *Falco peregrinus* hunt ECM on their approach to the roosting site and other predators such as Feral Cats *Felis catus* may take birds that roost closer to the ground^9^, but thermoregulatory demands seem the most likely factor separating the micro-sites. This could have important implications on energy conservation during a period when aerial insects are scarce. Certainly, differences in temperature between roosting sites of Cave Swallows *Petrochelidon fulva* have been suggested as an explanation for differences in condition of birds at different sites^25^.

The very high level of fidelity observed within and between years suggests that these patterns in use of micro-sites remain quite stable. As well as showing strong ties to particular micro-sites, the rate of recapture and proportion of birds recaptured more than once was highest at Gorham’s Cave and lowest at Vanguard Cave, the same pattern as that of body condition. The finding therefore reinforces the evidence that Gorham’s Cave provides better conditions as a micro-site for roosting. If winter mortality is an important driver in ECM population dynamics, micro-site selection could be an important factor in fitness of ECM. The finding helps to elucidate the value of these caves as winter refuges for this and other taxa.

A few birds were subsequently captured at different micro-sites from those where they had been first caught. The number was very small and this does not necessarily indicate that they were roosting at the new micro-site. For example, Sand Martins *Riparia riparia* regularly visit breeding colonies other than their own^26^ and it is possible that birds captured at a different micro-site were merely visitors that would nevertheless have returned to their own micro-sites to roost, especially when considering the proximity of these to each other within a single roosting site. However, a certain level of interchange can also be expected if micro-sites differ in their quality and birds in better condition can compete favorably for better sites. Under such a scenario, the population roosting in the micro-site with the higher proportion of birds in a good condition can be expected to be more stable than others. This is indicated by our results, which suggest that birds first captured in Gorham’s Cave showed even less mobility between micro-sites than those first captured at the other two caves. A third possibility is that fidelity to micro-sites is lower among immature birds^18,27^. Our incomplete ageing data could support this idea, but in any case, age of birds and competition for better micro-sites may not be independent factors, because adult ECM wintering at Gibraltar are larger and heavier than juveniles^13^. Incidental observations of birds chasing each other off ledges (*pers. obs.*) reinforces the likely role of competition in assortment of birds across micro-sites.

The wintering population of ECM at Gibraltar was assumed to have decreased markedly in recent times^5^, but our results suggest that numbers of ECM wintering at the Gorham’s Cave Complex remain comparable to those recorded during the 1970s, or perhaps exceed them. Some 1500 ECM have been recorded at a winter roost in Benicàssim on the Valencian coast of Spain^28^ but at its peak, the Gibraltar roost is much the largest on record and could be the largest globally, holding perhaps ~ 1–2% of the European post-breeding population of the species (based on^29^). Although the species’ conservation status is currently listed as ‘Least Concern’^30^, the scale of the aggregation illustrates the vulnerability that even such species may have to contingent events. The status of the Gorham’s Cave Complex as a UNESCO World Heritage Site, located within the Gibraltar Nature Reserve, helps to ensure the conservation of this important wintering population, which is as much a part of the site’s Pleistocene past as its present.

### The site

The Rock of Gibraltar holds a winter roosting site of ECM at the Gorham’s Cave Complex (Fig. 1), on its eastern side, first documented by John White during the eighteenth Century^31^. The size of the roost is large and was estimated at 1500–3100 in the 1970s^12,13^. Wintering ECM arrive in Gibraltar in early October and most leave in early March^9,12^. They leave the roost each day to forage to the north in nearby Spain, returning to the caves in the evening *en masse*, along a single trajectory^9,12,13^. The site is geomorphologically complex, with a series of eight caves and numerous cavities that lie side by side at sea level. They were formed by a combination of tectonic and eustatic processes^3^ and span some 300m along a north-south axis. All of these caves and cavities are used by roosting ECM, allowing the identification of micro-sites (caves and cavities) within the roost that are in close proximity to each other. Aerial insects are a scarce resource in temperate environments during the winter months^32^ and any factor influencing body condition could impact survivorship of an aerial insectivore wintering in the Western Palaearctic, including selection of wintering and roosting sites. This, combined with the site’s morphology, makes the Gorham’s Cave Complex an ideal site at which to study micro-site selection ecology in ECM.

## Methods

We carried out monitoring of ECM wintering at a roost in Gibraltar that consists of a series of caves alongside each other at sea level, primarily during the autumn–winter period of 2019-2020. The monitoring consisted of weekly counts of birds returning to roost, and of regular ringing and measuring sessions. The ringing data for 2019–2020 were augmented with data collected at the site between 2016 and 2018.

**Figure 4:**
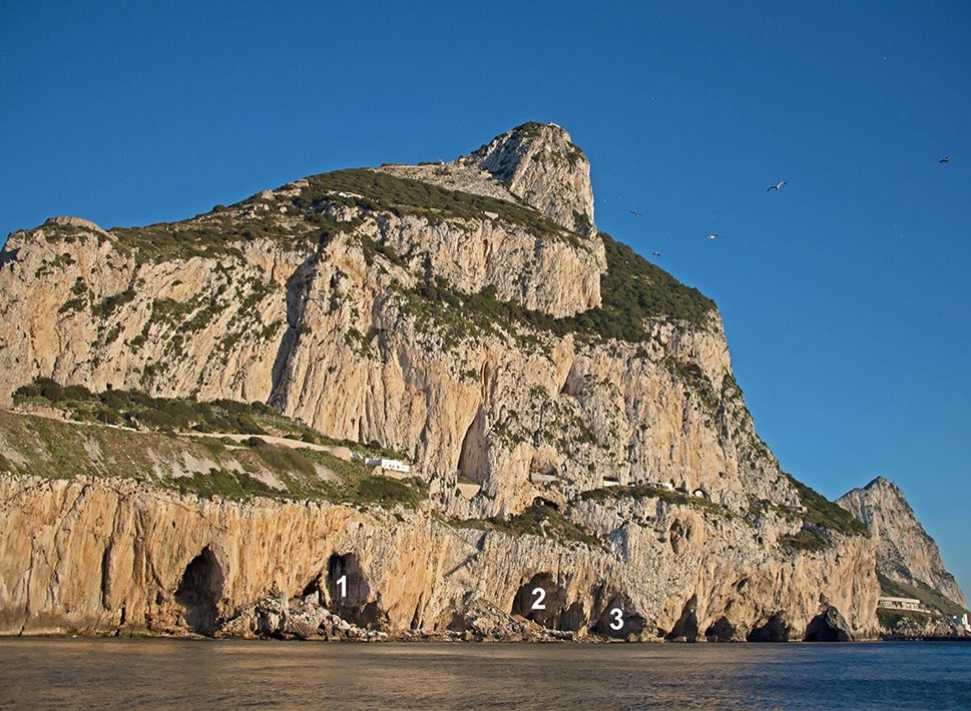
The position of Gorham’s Cave (1), Vanguard Cave (2) and Cave F (3) at the Gorham’s Cave Complex UNESCO World Heritage Site.

**Figure 5:**
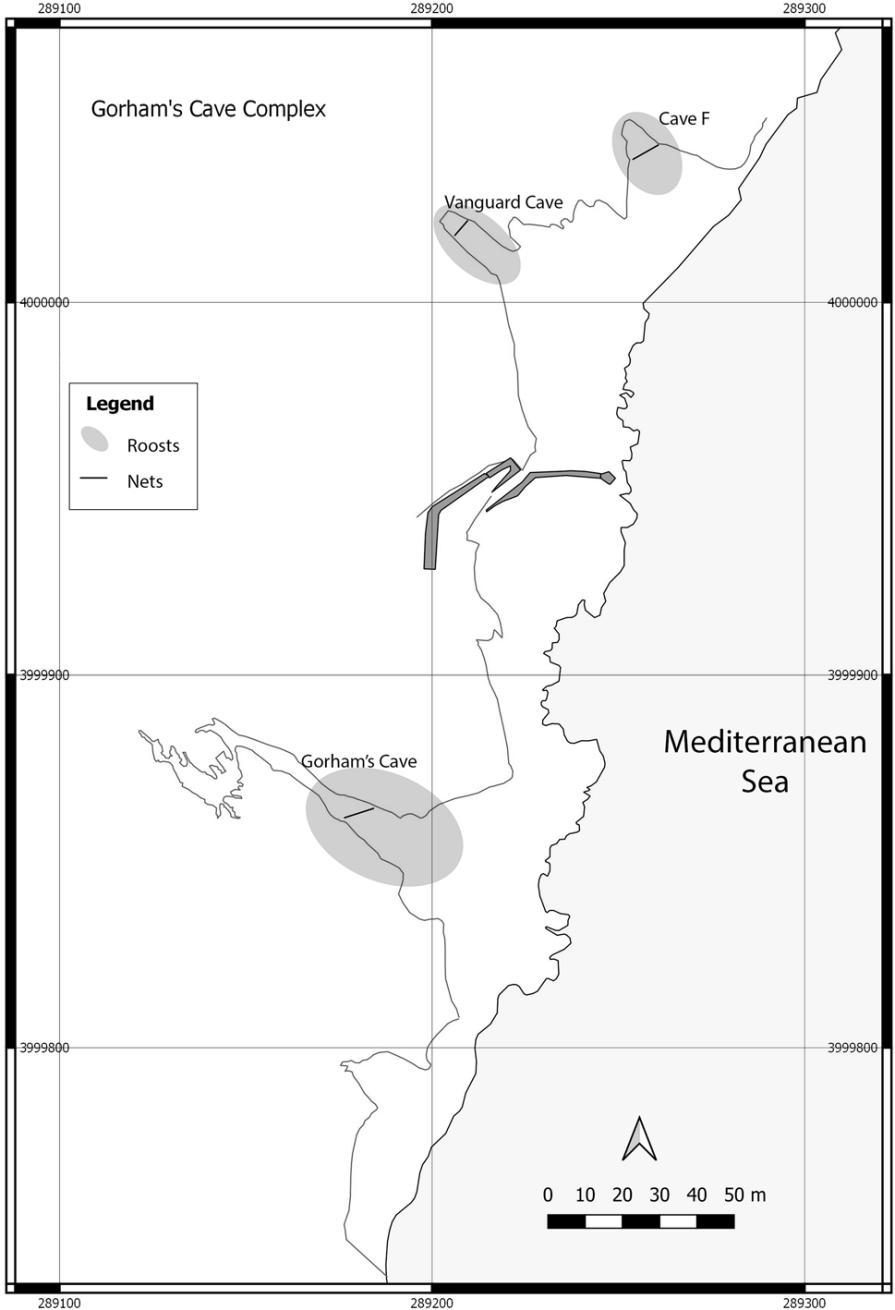
Plan map of Gorham’s Cave, Vanguard Cave and Cave F at the Gorham’s Cave Complex UNESCO World Heritage Site, indicating key roosting areas for ECM and the position of the mist nets at each cave.

The eight caves at the site are all micro-sites within a single ECM roost and three of them, which lie just above the current sea level and are the only ones accessible from land, were studied: Gorham’s Cave (36° 07′ 13.86″ N 5° 20′ 32.57″ W UTM 30 N 289190.5 3999856.5), Vanguard Cave (36° 07′ 18.89″ N, 005° 20′ 31.62″ W UTM 30 N 289218.0 4000011.0) and Cave F (36° 07′ 19.8″ N, 005° 20′ 30.088″ W UTM 30 N 289257.0 4000038.1) (Figs. 4 and 5).

Twenty-three weekly evening counts were conducted of birds returning to the roosting site during the period 4th October, 2019 to 12th March, 2020, from a fixed point overlooking the study site. We attempted to space these evenly in time, but adjustments were made due to unfavorable weather (mean number of days between counts = 6.96 ± 1.45 SE). The approach of the birds as they return to roost is described elsewhere^12^. It occurs along a fixed trajectory and our vantage point optimized the viewing of these movements. All ECM returned to the site along a common trajectory and birds only broke up and headed towards the different caves once at the site, so that in principle, every bird had equivalent opportunities to access each cave on arrival to the site.

We used a combination of the results of the counts and the Schnabel Index for mark-release-recapture data from a series of dates^33^ to estimate the total roosting population size of ECM at the site during the 2019–2020 period. The latter was achieved by estimating the number of birds roosting at each cave and then combining these for a total population size, although we recognize that birds also use other micro-sites^7^; (*pers. obs*.). Due to differences in sample sizes of birds recaptured, 95% confidence limits for the estimated roosting population size at each cave were drawn from the *t*-distribution for Gorham’s Cave and the Poisson distribution for Vanguard Cave and Cave F^33^.

Trapping and ringing were carried out at the three caves at least once a week. All licences required under the laws of Gibraltar were obtained and protocols were approved by the Ethics Committee of the University of Gibraltar. Ringing and handling of birds was carried out under the auspices of the Gibraltar Ornithological & Natural History Society (GONHS), which carries out its bird ringing under licence from the Ministry for the Environment, HM Government of Gibraltar, under the 1991 Nature Protection Act. Gibraltar-based ringers are licensed by the British Trust for Ornithology (BTO), and we adhered closely to the technical and ethical standards of the BTO for handling and ringing birds^34^. Routinely, birds are released without ringing when their condition is poor. One bird was captured in a condition that was too poor for it to be ringed. The reporting recommendations of the ARRIVE guidelines^35^ were followed.

The majority of the data used in this study were collected between October 29th 2019 and March 4th 2020. In addition, trapping and ringing had taken place intermittently at Vanguard and Cave F during the winter since 2016, and trapping took place at the site throughout autumn 2020. We used the BTO A-sized rings, in accordance with guidelines for other European hirundines^34^. Due to the different dimensions of the caves, we used different mist net sizes at each one. A 6m-length net was used at Vanguard Cave, 12 m and 3 m nets at Cave F, and 3 × 6 m nets mounted vertically on triple high poles at Gorham’s Cave.

The number of trapping sessions, and the range of dates of these at each cave during the 2019–2020 autumn-winter season, was: 10 Gorham’s Cave (29/10/2019–04/03/2020; mean number of days between sessions 14.11 ± 2.23 SE), 11 Vanguard Cave (13/11/2019–04/03/2020; mean number of days between sessions 11.20 ± 1.81 SE), 11 Cave F (13/11/2019–04/03/2020; mean number of days between sessions 11.20 ± 1.81 SE). Seven extra trapping sessions took place at Vanguard Cave and Cave F before the 2019-2020 autumn-winter season, on: 01/28/2016, 02/16/2016, 02/13/2018, 02/21/2018, 12/04/2018, 01/08/2019 and 02/21/2019. There were eight additional trapping sessions during the autumn of 2020, on: 10/29/2020, 11/02/2020, 11/12/2020, 11/15/2020, 11/19/2020, 11/24/2020, 12/02/2020 and 12/03/2020. 1511 different birds were processed between 2016–2020, of which 156 were captured at least twice. 796 individuals were processed during the 2019–2020 autumn-winter season, the period for which most of our analyses are based: 369 at Gorham’s Cave, 221 at Vanguard Cave and 206 at Cave F. Of the birds recaptured that had been ringed at the site during previous seasons, eighteen were from the 2019–2020 season (ten ringed at Gorham’s Cave, two at Vanguard Cave, seven at Cave F), fifteen were from the 2018–2019 season (eight at Vanguard Cave, six at Cave F), four were from the 2017–2018 season (three at Vanguard Cave, one at Cave F), and one was from the 2015–2016 season (from either Vanguard Cave or Cave F; unspecified and excluded from the analysis). A bird was recaptured that had been ringed elsewhere in Gibraltar (the GONHS Jews’ Gate Field Centre) on the 14/01/2014, 2233 days before it was captured again on the 25/02/2020.

Biometric measurement of all birds was carried out by a single person (CP) in order to maximize consistency. We followed the standard processing procedure of the BTO^34^, which includes recording the weight of birds in grams (g) to 0.1 g and length of wing in millimeters (mm) to 0.5 mm. Birds were aged whenever this was possible but ageing of ECM became increasingly difficult towards the end of the winter period, increasing the possibility of confusion with adults^9^. For this reason, age was excluded from most of the analyses. Birds could not be sexed because sexes are similar in appearance, including size^4,36^. We captured birds only during the evening, to ensure that condition of birds was not a factor of weight-loss whilst roosting, since ECM at the site are known to weigh less during mornings than the evenings^13^. Birds captured were roosted in boxes and released at the site the following morning.

Although Elkins & Etheridge^12^ assumed that movement of birds between different parts of the roost at Gibraltar is considerable, this was never tested. The proximity of different parts of the roost from each other means that all micro-sites are potentially equally accessible to ECM using the site. It is expected that they should be able to use micro-sites interchangeably, given especially their approach during evenings along a fixed narrow route. Any fidelity to micro-sites must thus be explained by factors other than distance between individual micro-sites. The multiple cavities at the roosting site, and the ease with which we were able to access these, allowed a unique opportunity to test whether individual birds repeatedly used the same micro-sites within the roost, both within and between winters. We used the data gathered to test the following hypotheses: (1) that a degree of fidelity to different spaces within the roost (‘micro-sites’) exists among ECM, with individuals more likely to be recaptured at the same cave than in a different cave, (2) that any fidelity observed will translate to a difference in quality of roosting sites, as indicated by differences in condition of birds according to micro-site, and (3) that the incidence of recapture should be highest at the cave at which birds are in the best condition.

Statistical analyses followed Sokal & Rohlf^37^ and were carried out on SPSS statistical software (IBM). We used a binomial *Z* test to analyze whether recaptured birds that were initially ringed during the 2019–2020 season were returning to the cave where they were first trapped/ringed, (1) within the 2019–2020 season and (2) between this and separate seasons. We also used a 3 × 2 Fisher’s exact test to test for differences, between caves, in the frequency with which birds ringed at one cave were captured at another. Multiple recaptures of birds were excluded from all of these analyses on fidelity in order to avoid bias.

We explored the relationship between wing length and weight using linear regression analysis, to control for the possible effect of body size on weight—on the basis that wing length provides a good measure of body size in passerines^38^—using only data collected during the 2019–2020 season. For individual birds that were trapped more than once, we used wing length and weight on the date of first capture. We then grouped, by cave, the residuals of the regression and used a one-way ANOVA to explore differences in mean condition of birds between caves, with condition expressed as the relationship between wing length and weight. We also used linear regression to explore the relationship between daily recapture rate at all caves and the number of days from the first day of trapping at each cave, with the latter as the explanatory factor. Again, we segregated the residuals of the regression by cave and used a one-way ANOVA to explore differences in recapture rates between caves. We used Pearson’s chi-squared test with Yates’s correction for small sample sizes^39^ to explore differences in the likelihood of recapture of birds on more than one occasion at each cave. Because differences in weight and wing length have been recorded between adult and juvenile ECM in Gibraltar^13^, we used Pearson’s chi-squared test to explore the relationship between age and use of the different micro-sites for all the birds that we were able to age (n = 395 of 796 birds processed), to see whether this was consistent with our other findings.

## Data Availability

All ringing and associated biometric data are deposited with the BTO. The datasets generated and analysed during the current study are available from the corresponding author on reasonable request.
